# Supplementary material for: In Vitro Anthelmintic Activity of a Hydroalcoholic Extract from Guazuma ulmifolia Leaves against Haemonchus contortus
Source: Pathogens. 2022 Oct 7;11(10):1160. doi: 10.3390/pathogens11101160 (PMC9607474; doi:10.3390/pathogens11101160)
Supplement: Supplementary file 1 [file pathogens-11-01160-s001.zip › pathogens-1903506-supplementary.pdf]

pathogens

MDPI

Supplementary data

In vitro Anthelmintic Activity of a Hydroalcoholic Extract from *Guazuma ulmifolia* Leaves Against *Haemonchus contortus*

Guillermo Reséndiz-González[1], Rosa Isabel Higuera-Piedrahita[2], Alejandro Lara-Bueno[1*], Roberto González-Gardúño[1], Jorge Alberto Cortes-Morales[3], Manases González-Cortazar[4], Pedro Mendoza-de Gives[5], Sara Guadalupe Romero-Romero[5] and Agustín Olmedo-Juárez[5*]

[1] Posgrado en Producción Animal, Departamento de Zootecnia, Universidad Autónoma Chapingo, Texcoco CP 56230, México
[2] Facultad de Estudios Superiores Cuautitlán, UNAM, Carr. Cuautitlán-Teoloyucan km 2.5 Col. San Sebastián Xhala. Cuautitlán. Estado de México, México
[3] Laboratorio de Fitoquímica y Productos Naturales. Centro de Investigación en Biodiversidad y Conservación. Universidad Autónoma del Estado de Morelos, Av. Universidad 1001, Colonia Chamilpa C.P. 62209, Cuernavaca, Morelos, México
[4] Centro de Investigación Biomédica Del Sur, CIBIS, IMSS, Argentina No. 1, Xochitepec, Morelos, México
[5] Centro Nacional de Investigación Disciplinaria en Salud Animal e Inocuidad, INIFAP, Morelos, Carr. Fed. Cuernavaca-Cuautla No. 8534, Jiutepec, Morelos, México
* Correspondence: authors: aolmedoj@gmail.com (A.O.J.) and alarab_11@hotmail.com (A.L.B.)

Citation: Reséndiz-González, G.; Higuera-Piedrahita, R.I.; Lara-Bueno, A.; González-Gardúño, R.; Cortes-Morales, J.A.; González-Cortazar, M.; Mendoza-de Gives, P.; Romero Romero, S.G.; Olmedo-Juárez, A.; *Pathogens* **2022**, *11*, x. https://doi.org/10.3390/pathogens11101160

Academic Editor: Elias Papadopoulos

Received:
Accepted:
Published:

Publisher's Note: MDPI stays neutral with regard to jurisdictional claims in published maps and institutional affiliations.

Copyright: © 2022 by the authors. Submitted for possible open access publication under the terms and conditions of the Creative Commons Attribution (CC BY) license (https://creativecommons.org/licenses/by/4.0/).

Content

Figure S1. HPLC chromatogram and UV spectra of the hydroalcoholic extract (HA-E) from *Guazuma ulmifolia* leaves. NI= No identified
Figure S2. HPLC chromatogram and UV spectra of the aqueous fraction (Aq-F) from *Guazuma ulmifolia* leaves. NI= No identified
Figure S3. HPLC chromatogram and UV spectra of the ethyl acetate fraction (EtOAc-F) from *Guazuma ulmifolia* leaves. NI= No identified

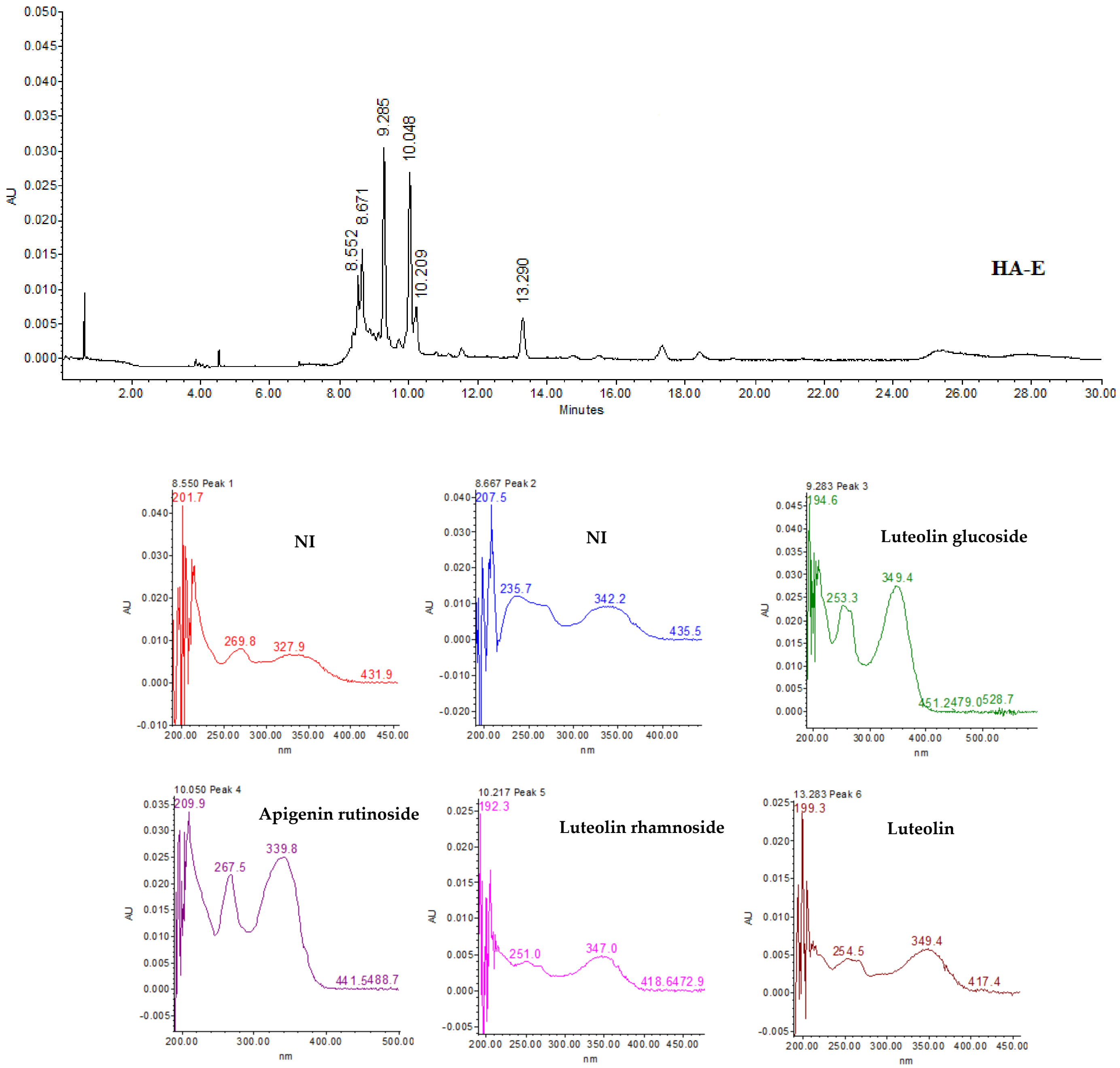

Figure S1. HPLC chromatogram and UV spectra of the hydroalcoholic extract (HA-E) from *Guazuma ulmifolia* leaves. NI= No identified.

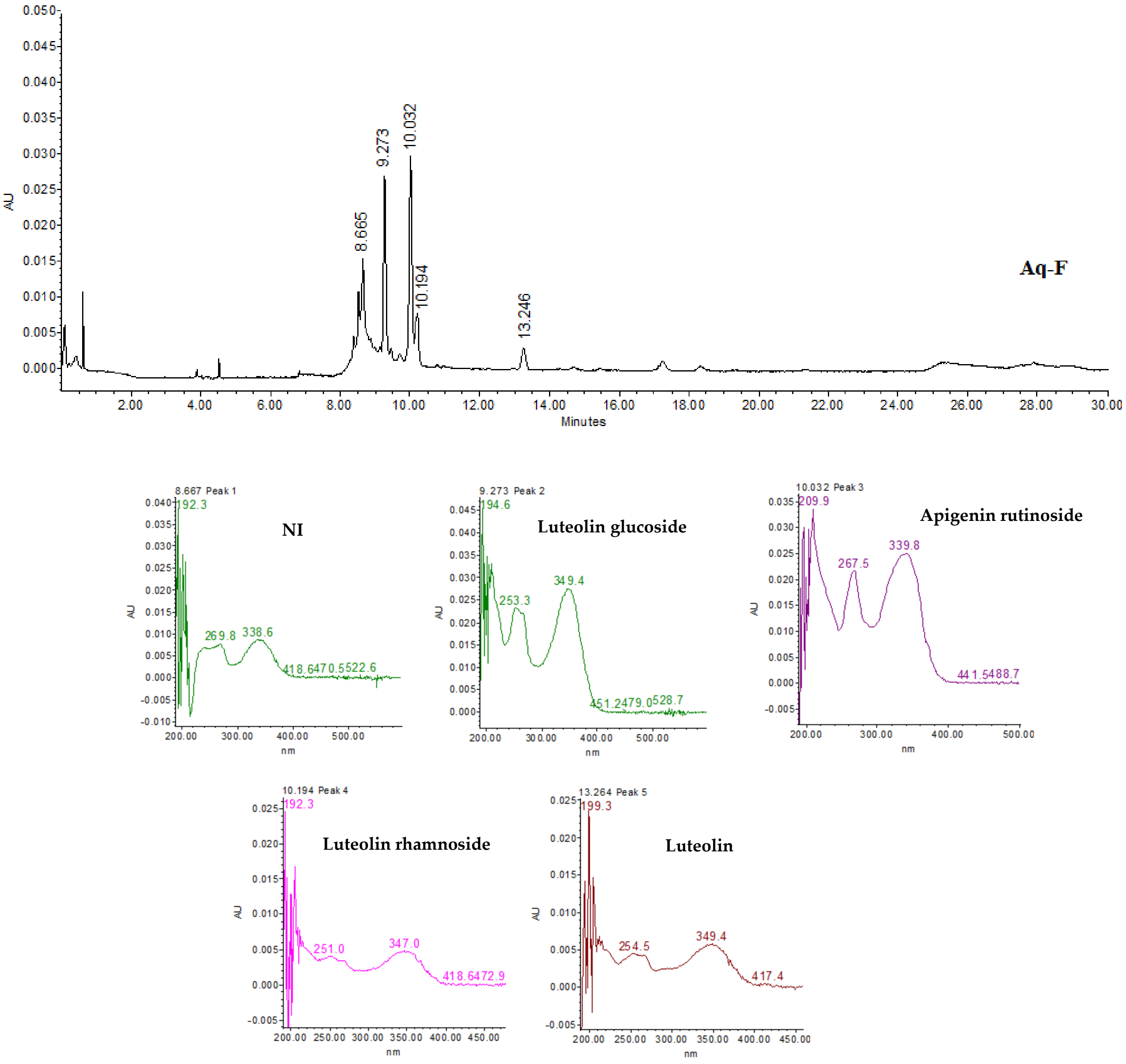

Figure S2. HPLC chromatogram and UV spectrum of the aqueous fraction (Aq-F) from *Guazuma ulmifolia* leaves. NI= No identified.

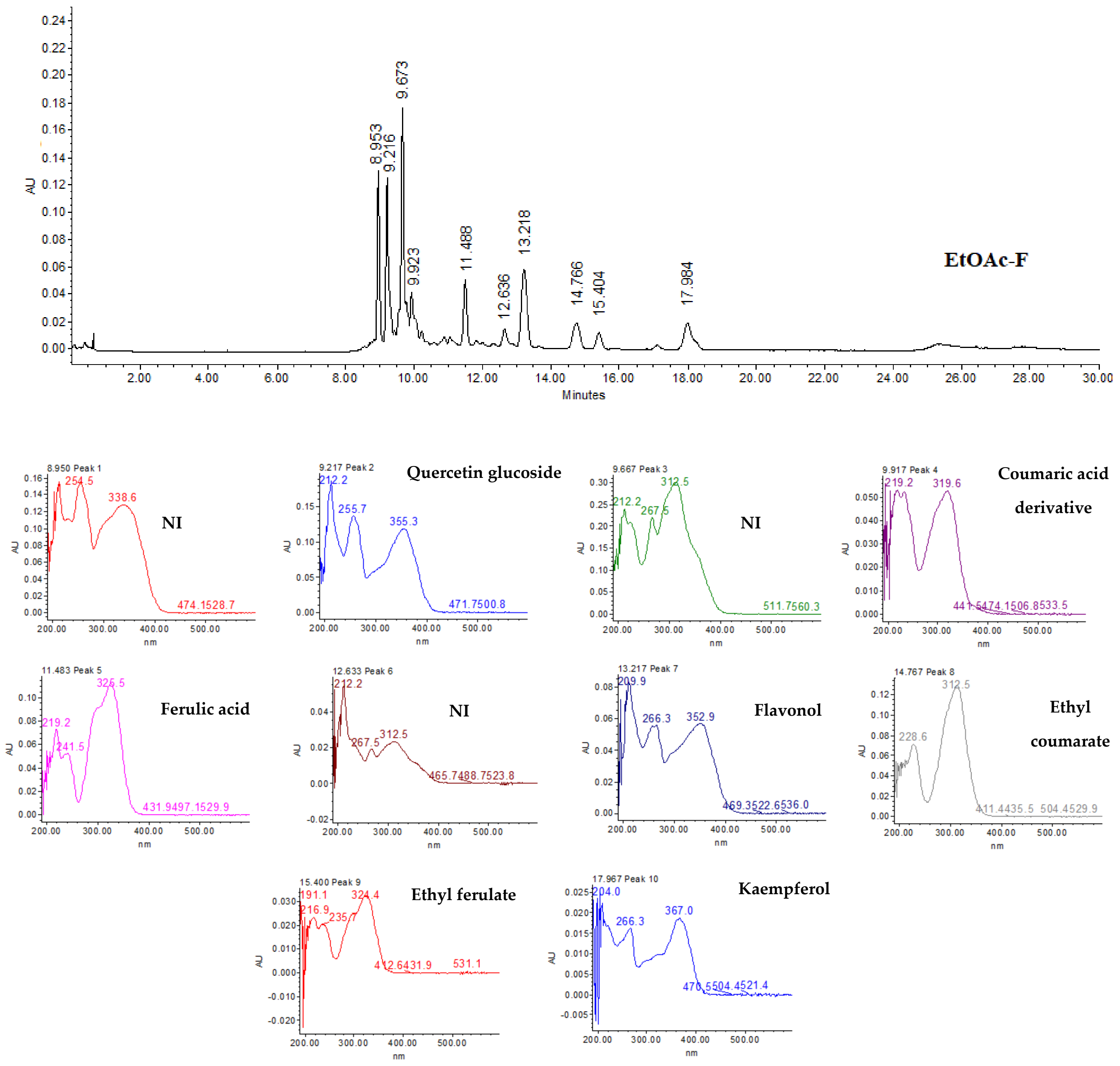

Figure S3. HPLC chromatogram and UV spectra of the ethyl acetate fraction (EtOAc-F) from *Guazuma ulmifolia* leaves. NI= No identified.
